# Expert assessment of the attributes of high-cost medications: a case study of CAR T-cell therapy in the gulf cooperation council

**DOI:** 10.3389/fphar.2026.1812612

**Published:** 2026-09-04

**Authors:** Yazed AlRuthia, Sara Al Dallal, Anas Hamad, Mohamed Ebrahim Al Khalifa, Sultana Al-Sabahi, Said Wani, Abdullah Alibrahim, Ibtisam Alharbi, Abdulaziz Alhenaidi, Mohammad Almari, Abdulrahman Aloumi, Mariam Aljalahma

**Affiliations:** 1 Pharmacoeconomics Research Unit, Department of Clinical Pharmacy, College of Pharmacy, King Saud University, Riyadh, Saudi Arabia; 2 Emirates Health Economics Society, Dubai, United Arab Emirates; 3 Drug Supply Department, Hamad Medical Corporation, Doha, Qatar; 4 College of Pharmacy, QU Health Sector, Qatar University, Doha, Qatar; 5 Health Strategic Assessment Directorate, Supreme Council of Health, Manama, Bahrain; 6 Ministry of Health, Muscat, Oman; 7 Industrial and Management Systems Engineering, College of Engineering and Petroleum, Kuwait University, Shadadiya, Kuwait; 8 Ministry of Defense Health Services, Riyadh, Saudi Arabia; 9 Director of Health Insurance Directorate, Ministry of Health, Sulaibikhat, Kuwait; 10 Department of Health Policy and Management, College of Public Health, Kuwait University, Safat, Kuwait; 11 Pharmaceutical Pricing Department, Medicine and Medical Products Registration and Regulatory Administration, Ministry of Health, Sulaibikhat, Kuwait; 12 CEO of Government Hospitals, Manama, Bahrain

**Keywords:** CAR T-cell therapy, conjoint analysis, gulf cooperation council (GCC), health policy, high-cost medications, managed entry agreements, market access, value-based healthcare

## Abstract

**Background:**

The influx of high-cost advanced therapies, particularly CAR T-cell therapies, poses profound economic and operational challenges for healthcare systems in the Gulf Cooperation Council (GCC). As the region transitions from volume-based procurement to Value-Based Healthcare (VBHC), decision-makers must balance the urgent need for innovative curative treatments with fiscal sustainability and limited local health economics data.

**Methods:**

This study utilized a quantitative expert preference rating approach, using a rating-based Conjoint Analysis to assess the utility and relative importance of key value drivers for CAR T-cell therapies. Eighteen Key Opinion Leaders (KOLs) evaluated four hypothetical CAR T-cell product profiles. Individual ratings for sequentially presented attributes were collected from participants and subsequently analyzed using an ordinary least squares (OLS) transformation regression model based on Multi-Attribute Utility Theory (MAUT) to estimate part-worth utilities.

**Results:**

The analysis identified labeled indications and outcome-based agreements as the dominant drivers of decision-making utility, with markedly higher estimated utility values than upfront price and individual efficacy or safety metrics. Specifically, a cohort-level Outcome-Based Agreements model, where reimbursement is conditional on the response rate of a treated patient population, generated the single highest utility value (U = 24.07). In terms of structural attributes, experts demonstrated a statistically significant preference for comprehensive Patient Support Programs (PSPs) and Technology Transfer commitments (local manufacturing) over simple scientific guidance or basic discounts.

**Conclusion:**

Decision-makers in the GCC prioritize risk-sharing mechanisms and broad therapeutic indications over traditional cost-containment strategies when evaluating high-cost CAR T-cell therapies. These findings suggest that to ensure equitable and sustainable patient access, policymakers must establish unified regulatory frameworks that facilitate performance-linked reimbursement models and incentivize the localization of advanced therapy infrastructure.

## Introduction

The rapidly evolving landscape of modern pharmacology has been fundamentally transformed by the advent of advanced therapies, including gene therapies and cell-based immunotherapies (such as CAR T-cell therapies). While the European Medicines Agency (EMA) legally classifies these as Advanced Therapy Medicinal Products (ATMPs), the Gulf Cooperation Council (GCC) regulatory bodies increasingly evaluate these high-cost interventions under specialized, though sometimes fragmented, regulatory pathways (Hanna et al., 2016). This study focuses on CAR T-cell therapies, which demonstrate significant potential for treating intractable hematologic malignancies by producing durable responses and, in certain cases, potentially curative outcomes after a single administration. However, these therapies also present substantial economic and operational challenges. These therapies pose a dual challenge for payers: substantial upfront costs ($200,000 to $500,000 per patient) and persistent uncertainty regarding long-term effectiveness and safety (Iancu and Kandalaft, 2020; Wenzl and Chapman, 2019; Sacr et al., 2022). The deployment of these complex treatments further necessitates rigorous technical transfer processes and specialized institutional support to ensure the maintenance of product quality and safety standards during manufacturing and administration (Iancu and Kandalaft, 2020). To address the financial risks associated with their high upfront costs and narrow labeled indications, health systems are increasingly adopting managed entry agreements (MEAs), encompassing both outcome-based and financial-based agreements, to link reimbursement to real-world therapeutic performance and budget impact (Wenzl and Chapman, 2019). Concurrently, pharmaceutical manufacturers have implemented patient-support programs (PSPs) to mitigate out-of-pocket expenditures and facilitate patient access. However, these initiatives often obfuscate accurate transaction prices and complicate value assessment (Sacr et al., 2022). Crucially, these economic barriers are exacerbated in low- and middle-income countries (LMICs), where constrained healthcare budgets, lack of infrastructure, and insufficient regulatory frameworks create a stark divide in the affordability and accessibility of these life-saving therapies compared to high-income nations (Ocran Mattila et al., 2021).

In the specific context of the Arabian Gulf Cooperation Council (GCC) countries, comprising Saudi Arabia, the UAE, Kuwait, Qatar, Oman, and Bahrain, the adoption of high-cost advanced therapies is occurring against a backdrop of urgent economic transformation, as healthcare systems strive to transition from passive, volume-based procurement to sustainable, value-based healthcare (VBHC) frameworks amidst fluctuating oil revenues (Hamad et al., 2025; AlRuthia et al., 2025). Recognizing the financial untenability of paying upfront for potentially curative, high-cost treatments, Saudi Arabia has emerged as a regional pioneer by actively engaging in outcome-based agreements (OBAs) and financial-based risk-sharing models for high-cost gene therapies and Chimeric Antigen Receptor (CAR) T-cell therapy therapies, such as *tisagenlecleucel* and *onasemnogene abeparvovec*, thereby linking reimbursement directly to real-world clinical success and patient survival (Al-Omar et al., 2025; Abu-Shraie et al., 2023). However, the broader implementation of such complex agreements across the GCC is frequently stifled by significant structural barriers, including the lack of a unified legal framework for risk-sharing and the absence of integrated electronic health records (EHRs) needed to generate the real-world evidence (RWE) required to track long-term patient outcomes reliably (Hamad et al., 2025; AlRuthia et al., 2025; Abu-Shraie et al., 2023). Furthermore, decision-makers in the region frequently struggle with insufficient local expertise in health economics and outcomes research (HEOR), often forcing a reliance on health technology assessment (HTA) reports from foreign bodies that may not reflect local cost structures, disease burdens, patient demographics, or local patient preferences (Hamad et al., 2025; Abu-Shraie et al., 2023). Operational hurdles also abound, as the technical transfer of complex manufacturing processes and rigid procurement policies, even in resource-rich nations like the GCC, can create budgetary silos that delay patient access and compromise the quality of care (Iancu and Kandalaft, 2020; Mirza et al., 2023). Consequently, despite the political will to adopt innovation, these barriers highlight the critical need for a unified regulatory framework and enhanced regional collaboration to harmonize labeled indications, streamline pricing strategies, and ensure equitable and financially sustainable access to these life-altering medicines across the Gulf (Hamad et al., 2025; Al-Omar et al., 2025). The urgency of this challenge is intensifying. The GCC approved its first CAR T-cell therapies in 2019–2020, with patient volumes growing from dozens to hundreds annually (Hashmi et al., 2024). Yet decision-making remains largely opaque, with limited published evidence on what drives formulary inclusion beyond anecdotal reports of individual payer negotiations. This study provides the first systematic, quantitative assessment of GCC decision-maker preferences.

Given the identified gaps in local health economics data and the increasing pressure to adopt value-based assessment models in the region (Hamad et al., 2025), the primary objective of this study was to quantitatively assess the utility and relative importance of key decision-making attributes associated with high-cost CAR T-cell therapies from the perspective of Key Opinion Leaders (KOLs) in the GCC. Specifically, this research aimed to evaluate how decision-makers weigh clinical outcomes against economic and structural factors when selecting therapies for reimbursement. Ultimately, by disaggregating these preference weights, this research intends to provide empirical evidence to support the development of a unified, evidence-driven framework for the sustainable adoption of CAR T-cell therapies across GCC healthcare systems.

## Methods

### Study design and setting

This study utilized an expert-driven approach to assess the value attributes of CAR T-cell therapies. The research employed a rating-based conjoint analysis (RBCA) model centered on CAR T-cell therapies, which are classified as high-cost advanced therapies within the GCC region. The expert workshop took place in Dubai, United Arab Emirates, on 4 October 2025.

The Emirates Health Economics Society organized this session in partnership with the Gulf Health Economics Association. The main goal was to gather expert opinions and establish the utility weights for various clinical, economic, and structural attributes related to the implementation of high-cost cellular therapies.

### Participants

#### Participant demographics and characteristics

Participants were identified through purposive sampling using the professional networks of the Emirates Health Economics Society and the Gulf Health Economics Association. Eligibility criteria included only individuals with documented involvement in national or institutional health technology assessment (HTA), ensuring the sample’s relevance. In the decentralized GCC formulary context, eligible decision-makers were defined as clinical leaders, health policy experts, and procurement directors who advise on or directly authorize the adoption of high-cost therapeutics.

Potential participants received formal email invitations outlining the study’s objectives. In total, 25 individuals were initially invited to participate. Our original goal was to recruit a minimum sample size of 12 Key Opinion Leaders (KOLs), targeting 2–3 participants per GCC member state. In total, 18 of the 25 invited participants completed the study, yielding a response rate of 72.0%.

The final panel of 18 experts included representation from all six GCC countries, comprising participants from Kuwait (27.8%), Oman (22.2%), Bahrain (16.7%), the UAE (16.7%), Qatar (11.1%), and Saudi Arabia (5.6%). Furthermore, the sample captured a wide cross-section of health-system settings, with experts affiliated with regulatory health authorities (55.6%), academia (22.2%), public healthcare institutions (11.1%), and private healthcare institutions (11.1%). The resulting multidisciplinary cohort primarily consisted of health economists (33.3%), pharmacists (33.3%), and physicians (22.2%), with half of the participants currently holding positions directly related to pharmaceutical planning and purchasing. Over half (55.6%) held a PhD or equivalent degree. Detailed demographics are provided in Table 1.

**TABLE 1:** Participants’ characteristics (n = 18).

Characteristic	n (%)
Gender	
Male	12 (66.7)
Female	6 (33.3)
Country	
Kingdom of Bahrain	3 (16.7)
Kingdom of Saudi Arabia	1 (5.6)
Kuwait	5 (27.8)
Qatar	4 (11.1)
United Arab Emirates (UAE)	3 (16.7)
Sultanate of Oman	4 (22.2)
Affiliation	
Academia	4 (22.2)
Regulatory health authorities	10 (55.6)
Public healthcare institutions	2 (11.1)
Private healthcare institutions	2 (11.1)
Profession	
Health economist	6 (33.3)
Healthcare management specialist	2 (11.1)
Pharmacist	6 (33.3)
Physician	4 (22.2)
Age	
25–35 years	3 (16.7)
36–40 years	7 (38.9)
41–45 years	6 (33.3)
51–55 years	1 (5.6)
>60 years	1 (5.6)
Years of experience	
1–5 years	1 (5.6)
6–10 years	2 (11.1)
11–15 years	8 (44.4)
16–20 years	5 (27.8)
>20 years	2 (11.1)
Working in a position related to pharmaceutical planning and purchasing	
Yes	9 (50.0)
No	9 (50.0)
Member in the pharmacy and therapeutics committee	
Yes	6 (33.3)
No	12 (66.7)
Highest educational degree	
Bachelor degree or equivalent	2 (11.1)
Master degree or equivalent	6 (33.3)
PhD or equivalent	10 (55.6)

### Ethical considerations

The study protocol adhered to the Declaration of Helsinki and was approved by the Research Ethics Board of the Emirates Health Economics Society (Approval# EHEC-02) (World Medical Association Declaration of Helsinki, 2013). All participants provided written informed consent.

Before the meeting, a detailed information sheet outlining the study’s objectives was distributed to all invitees via email. All participants provided written informed consent before the workshop began. To ensure confidentiality, no personal identifiers, such as names, addresses, or identification numbers, were collected or retained. Instead, the data were aggregated and anonymized for subsequent analysis.

### Attribute selection

Attributes and their corresponding levels were identified through a targeted review of CAR T-cell therapies Health Technology Assessment (HTA) reports. The final selection comprised nine critical domains:

1. Efficacy (ORR and CR rates)
2. Safety (Incidence of CRS and ICANS)
3. Labeled Indications
4. Price
5. Patient Support Programs (PSPs)
6. Institutional Support
7. Technology Transfer
8. Outcome-Based Managed Entry Agreements (MEAs)
9. Financial-Based MEAs

The levels were validated through preliminary scoping discussions, including unstructured interviews with three regional health economists and two clinical pharmacists, who were separate from the final panel of 18 KOLs that completed the rating exercise. This process ensured that the levels reflected the real-world trade-offs encountered by GCC decision-makers. The “Technology Transfer” attribute was included in response to feedback indicating that localization is a major priority for GCC decision-makers (Bridges et al., 2011; Kløjgaard et al., 2012). The ‘Price’ attribute estimated the wholesale acquisition cost (WAC) per treatment in US dollars (USD) from the payer’s perspective, prior to the application of any subsequent outcome-based or financial-based rebates.

Given the specialized nature of RBCA and the relative scarcity of standardized reporting guidelines specific to this method in health economics, we utilized established Good Research Practices for conjoint analysis and Discrete Choice Experiments (DCE) to guide the methodological framework (Bridges et al., 2011; Coast et al., 2012).

### Rating-based CA

A Rating-Based Conjoint Analysis (RBCA) methodology was selected to manage the high cognitive burden associated with evaluating nine complex attribute domains (Arroyo et al., 2017). Unlike choice-based models, this compositional approach allows experts to evaluate the intrinsic value of multifaceted profiles sequentially, minimizing evaluation fatigue (Asioli et al., 2016). The theoretical foundation relies on multi-attribute utility theory (MAUT), where total utility is the additive sum of part-worths. The global rating for a specific product profile was derived as the sum of the ratings assigned to its constituent attributes, allowing for the isolation of value drivers specific to the GCC healthcare context (Baltussen and Niessen, 2006).

### Product profiles

Four hypothetical CAR T-cell therapy profiles (Products One through Four) were deliberately designed to capture the diverse variations in product characteristics currently present in the market for hematologic immunotherapies. The specific profiles and attribute combinations were directly informed by our targeted HTA literature review and the preliminary discussions with the five regional experts. To ensure methodological rigor, attribute levels were allocated across the four profiles to maintain balance and clinical plausibility, deliberately avoiding implausible combinations (e.g., pairing maximum efficacy with the lowest possible safety and structural support). These profiles were constructed to reflect a mix of existing market options and plausible future trade-offs. Attribute levels were combined to force participants to weigh differing priorities, such as high clinical efficacy against limited structural support, rather than perfectly mimicking specific commercial products currently available.

o Product One focused on broad indications and comprehensive PSPs.
o Product Two featured high efficacy but limited structural support.
o Product Three introduced cohort-level outcome-based agreements.
o Product Four represented a lower-priced option with localized manufacturing guidance.

Detailed levels for each profile were presented to the experts to ensure realistic market assessments.

The specific levels for each product were presented to the experts as follows:

### Indications and clinical efficacy

The products were evaluated based on their approved indications and efficacy data (Overall Response Rate [ORR] and Complete Response Rate [CR]) derived from pivotal Randomised Controlled Trials (RCTs).

- Target Diseases: The case study covered Relapsed/Refractory (R/R) B-cell Acute Lymphoblastic Leukemia (B-ALL), Large B-cell Lymphoma (LBCL), Multiple Myeloma (MM), Mantle Cell Lymphoma (MCL), and Follicular Lymphoma (FL).
- Indications:
o Product One: Indicated for B-cell precursor ALL, R/R LBCL, and R/R FL.
o Product Two: Indicated for R/R LBCL and R/R FL.
o Product Three: Indicated for R/R LBCL, R/R FL, R/R CLL/SLL, and R/R MCL.
o Product Four: Indicated for R/R LBCL and R/R Multiple Myeloma.

#### Safety profile

Safety was assessed via the incidence of Cytokine Release Syndrome (CRS) and Immune Effector Cell-Associated Neurotoxicity Syndrome (ICANS).

- CRS: Participants considered rates of severe CRS (Grade 3/4), which typically range from 1% to 23% in clinical data.
- ICANS: Participants considered neurological risks such as encephalopathy, aphasia, and cerebral edema.

### Patient support programs (PSP)

Participants evaluated the value-added services provided by the manufacturer to support patient access and adherence.

o Product One: Offered transportation for patients/caregivers, accommodation/food vouchers, and financial aid.
o Product Two: Focused on online educational materials and disease awareness programs.
o Product Three: Provided psychological support by covering expenses for certified clinical psychologists.
o Product Four: Provided one-to-one online educational sessions with certified health educators.

### Institutional support

This attribute defined the manufacturer’s support for hospital infrastructure and staff readiness.

- Product One and Two: Included training for staff certification, partial payment for Risk Evaluation and Mitigation Strategy (REMS) training, and assistance with adverse event (AE) reporting platforms (Product One) or emergency staff training (Product Two).
- Product Three: Focused solely on training emergency staff to manage AEs.
- Product Four: Limited to training for hospital/staff certification.

### Technology transfer and infrastructure

This attribute assessed the manufacturer’s commitment to localizing production capabilities.

- Local Manufacturing: Products One, Two, and Three offered to help establish a regional manufacturing site, contingent on a public health system commitment to treat a specific annual volume of patients (60, 280, and 230 patients, respectively).
- Guidance Only: Product Four offered scientific guidance for establishing a site but no direct investment or volume-based commitment.

### Managed entry agreements (MEA): outcome-based

Participants evaluated risk-sharing schemes tied to clinical outcomes. All products required a registry (Coverage with Evidence Development) to capture effectiveness at 12 and 18 months.

- Performance-Linked Payment:
o Product One: 54% initial payment; 46% conditional on individual patient response at 18 months.
o Product Two: 36% initial payment; 64% conditional on individual patient response at 18 months.
o Product Three: 50/50 split, conditional on **80%** of the treated cohort sustaining response.
o Product Four: 50/50 split, conditional on 50% or more of the treated cohort sustaining response.

### Managed entry agreements (MEA): financial-based

This attribute involved direct cost-containment strategies.

- Product One: Free treatment for one patient for every 10 treated.
- Product Two: Coverage for all eligible patients after an annual threshold of 25 patients is met.
- Product Three: A simple 25% discount if at least 10 patients are treated annually.
- Product Four: A flat 15% discount regardless of volume.

### Data collection procedure

During the workshop, descriptions of each attribute for the four products were presented sequentially via PowerPoint. Although conducted during an expert panel meeting, participants submitted independent, blinded evaluations using an electronic form; the results represent aggregate derived preferences rather than a forced unanimous consensus. Participants rated the perceived value of each attribute on a 10-point Likert scale (1 = lowest, 10 = highest).

### Statistical analysis

Data were analyzed using SAS version 9.4. An Ordinary Least Squares (OLS) regression framework was used to estimate utilities, as the theoretical foundation rests on Multi-Attribute Utility Theory (MAUT) rather than Random Utility Theory. Relative importance was calculated by normalizing the range of part-worth utilities within each domain to 100%.

Conjoint analysis was conducted using the Transformation Regression procedure. The 10-point preference rating served as the continuous dependent variable (identity transformation) and was regressed on the categorical attribute levels, which were modeled using effects coding (sum-to-zero). This approach produced part-worth utilities, represented by the derived beta coefficients for each attribute level, reflecting their independent contributions to the overall product rating. Within this regression framework, the reported p-values and 95% confidence intervals assessed the null hypothesis that an individual attribute level’s part-worth utility (beta coefficient) equals zero. Formal statistical contrasts between different attribute utility estimates or importance scores were not performed; thus, comparisons between attributes are presented descriptively based on their relative utility weights. Additionally, the standard ordinary least squares (OLS) model used in this exploratory analysis treated individual ratings as independent observations and did not adjust for within-respondent correlation resulting from multiple ratings provided by each of the 18 experts. Because effects coding was used, positive or negative utility values should be interpreted as preferences relative to the mean utility within that attribute domain, not as absolute measures of benefit.

Finally, to determine Attribute Importance, the relative importance of each attribute was calculated by determining the range of part-worth utilities (maximum minus minimum utility) within each respective attribute domain. These ranges were then summed and normalized to 100% to reflect the proportional weight of each attribute in the experts’ overall assessment of the drug’s value. Descriptive statistics (means and standard deviations) were also calculated for the raw ratings to characterize the preference levels among the GCC experts.

## Results

### Conjoint analysis of attribute utilities

The analysis revealed that Labeled Indications and Outcome-Based Agreements (OBAs) were the dominant drivers of decision-making utility. Specifically, a cohort-level OBA model generated the highest utility value (*U* = 24.07; 95% CI: 15.01 to 33.13; *p* < 0.0001). Table 2 summarizes the ratings, utility estimates, and 95% confidence intervals (CI).

**TABLE 2:** Mean ratings and derived part-worth utilities (beta coefficients) for CAR T-cell therapy attributes.

Product	Attribute	Description	Mean rating ± SD	Utility	95% CI		*p*-value
					Lower limit	Upper limit	
One	Efficacy	Achieved 35% complete response (CR) and 40% objective overall response rate (ORR)	4.89 ± 1.78	3.77	−5.95	13.50	0.4352
	Safety	Incidence of grade 3 or severe cytokine release syndrome (CRS) is 32%, and immune effector cell-associated neurotoxicity syndrome (ICANS) is 41.5%	3.78 ± 2.34	−9.15	−25.89	7.59	0.2737
	Labeled indications	1. B-cell precursor acute lymphoblastic leukemia (ALL) (up to 25 years) 2. Relapsed/Refractory (r/r) large B-cell lymphoma (adults, failed 2+ lines) 3. r/r follicular lymphoma (FL) (adults, 2+ lines) *Not indicated for primary CNS lymphoma*	5.72 ± 1.90	24.12	8.99	39.24	0.0027*
	Prices	$320,000 per treatment	4.94 ± 1.98	−8.56	−18.84	1.73	0.0998
	Patient support programs	1. Educational programs 2. Transportation for patients/caregivers 3. Accommodation/Food vouchers for caregivers 4. Financial aid for families in need	6.83 ± 2.33	17.11	7.69	26.54	0.0008*
	Outcome-based agreements	Coverage with evidence (CED): Registry to capture effectiveness at 12 and 18 months Performance linked: 54% upfront; 46% payment if patient sustains response at 18 months (individual level)	6.00 ± 2.33	−0.58	−9.95	8.78	0.8994
	Financial-based agreements	Free treatment for one patient for every 10 patients treated	4.83 ± 1.98	16.53	5.87	27.20	0.0035
	Institutional support	1. Certification training for hospital/staff 2. Partial payment for REMS training 3. Assist in establishing adverse drug events platform	7.28 ± 2.22	4.80	−4.97	14.58	0.3244
	Technology transfer	Help establish manufacturing site if public health system commits to 60+ patients annually	6.44 ± 2.66	11.60	4.49	18.71	0.0022
Two	Efficacy	Achieved 58% complete response (CR) and 83% objective overall response rate (ORR)	7.11 ± 1.84	−1.59	−11.91	8.73	0.7563
	Safety	Incidence of grade 3 or severe cytokine release syndrome (CRS) is 20%, and immune effector cell-associated neurotoxicity syndrome (ICANS) is 31%	4.44 ± 1.89	4.52	−6.19	15.23	0.3959
	Labeled indications	1. Large B-cell lymphoma (LBCL) 2nd line (refractory or relapse within 12 months) 2. LBCL 3rd line or later (after 2+ lines) 3. Follicular lymphoma (FL) (r/r after 2+ lines)	5.72 ± 1.90	12.02	−2.21	26.24	0.0950
​	Prices	$307,000 per treatment	5.72 ± 2.14	2.59	−9.04	14.24	0.6523
	Patient support programs	1. Educational programs 2. Access to online educational materials	4.39 ± 1.50	−3.48	−18.27	11.31	0.6348
	Outcome-based agreements	Coverage with evidence (CED): Registry to capture effectiveness at 12 and 18 months Performance linked: 36% upfront; 64% payment if patient sustains response at 18 months (individual level)	6.67 ± 2.68	1.06	10.33	8.21	0.8175
	Financial-based agreements	Committed to treat any eligible patient after an annual threshold of 25 patients	6.17 ± 1.76	10.11	2.17	18.04	0.0142
	Institutional support	1. Certification training for hospital/staff 2. Partial payment for REMS training 3. Training emergency staff to manage adverse events	6.67 ± 2.20	13.57	−1.49	28.64	0.0759
	Technology transfer	Help establish manufacturing site if public health system commits to 280+ patients annually	6.17 ± 2.81	8.23	0.34	16.11	0.0414
Three	Efficacy	Achieved 53% complete response (CR) and 73% objective overall response rate (ORR)	6.56 ± 1.85	6.63	3.65	16.91	0.1980
	Safety	Incidence of grade 3 or severe cytokine release syndrome (CRS) is 10%, and immune effector cell-associated neurotoxicity syndrome (ICANS) is 15%	5.89 ± 1.75	4.02	−4.84	12.88	0.3622
	Labeled indications	1. LBCL (refractory/relapse <12months OR ineligible for HSCT OR r/r after 2+ lines) 2. r/r chronic lymphocytic leukemia (CLL) or small lymphocytic lymphoma (SLL) (2+ lines inc. BTK/bcl-2) 3. r/r follicular lymphoma (FL) (2+ lines) 4. r/r mantle cell lymphoma (MCL) (2+ lines inc. BTK)	7.56 ± 2.28	4.52	−9.04	18.08	0.5024
	Prices	$401,000 per treatment	4.67 ± 1.68	−5.89	−17.02	5.22	0.2882
	Patient support programs	1. Educational programs 2. Psychological support (paying for certified clinical psychologists)	5.72 ± 1.67	1.79	−6.73	10.33	0.6709
	Outcome-based agreements	Coverage with evidence (CED): Registry to capture effectiveness at 12 and 18 months Performance linked: 50% upfront; 50% payment if 80% of treated patients sustain response at 18 months (cohort level)	6.28 ± 2.49	24.07	15.01	33.13	<0.0001
	Financial-based agreements	Simple discount of 25% provided that at least 10 patients are treated annually	5.72 ± 1.74	4.81	−2.88	12.49	0.2120
	Institutional support	Training the emergency staff to manage adverse drug events	4.78 ± 2.53	7.81	−0.212	15.83	0.0560
​	Technology transfer	Help establish manufacturing site if public health system commits to 230+ patients annually	6.50 ± 2.55	7.79	0.355	15.23	0.0406
Four	Efficacy	Achieved 33% complete response (CR) and 73% objective overall response rate (ORR)	5.28 ± 1.81	4.24	−4.71	13.19	0.342
	Safety	Incidence of grade 3 or severe cytokine release syndrome (CRS) is 7%, and immune effector cell-associated neurotoxicity syndrome (ICANS) is 9%	6.61 ± 2.06	1.35	−6.38	9.09	0.7238
	Labeled indications	Relapsed or refractory multiple myeloma (RRMM) who have received at least one prior line (inc. PI, IMID) and are refractory to lenalidomide. Targets BCMA protein	5.11 ± 1.84	11.03	2.78	19.28	0.0104*
	Prices	$255,000 per treatment	6.33 ± 2.22	−1.66	−9.66	6.34	0.6749
	Patient support programs	Trained/Certified health educators provide one-to-one online educational sessions	4.72 ± 2.05	1.67	−7.91	11.25	0.7249
	Outcome-based agreements	Coverage with evidence (CED): Registry to capture effectiveness at 12 and 18 months Performance linked: 50% upfront; 50% payment if 50% or more of treated patients sustain response at 18 months (cohort level)	6.00 ± 2.25	9.35	−0.09	18.79	0.0521
	Financial-based agreements	Simple discount of 15% regardless of the number of patients treated	5.39 ± 2.12	14.37	6.19	22.56	0.0011
	Institutional support	Training to help the hospital and its staff get certified to administer this treatment	4.39 ± 2.64	6.99	−5.54	19.54	0.2641
	Technology transfer	Provide scientific guidance only for establishing a manufacturing site	4.72 ± 2.52	−1.24	−9.82	7.33	0.7698

* CR: complete response, ORR: objective overall response rate, CRS: cytokine release syndrome, ICANS: Immune Effector Cell-Associated Neurotoxicity Syndrome, ALL: acute lymphoblastic leukemia, LBCL: Large B-cell Lymphoma, FL: follicular lymphoma, CNS: central nervous system, REMS: risk evaluation and mitigation strategy, CED: Coverage with Evidence Development), CLL: chronic lymphocytic leukemia, SLL: small lymphocytic lymphoma, MCL: mantle cell lymphoma, RRMM: relapsed or refractory multiple myeloma, PI: proteasome inhibitor, IMID: immunomodulatory imide drug, BCMA: B-cell Maturation Antigen.

### Clinical attributes: indications, efficacy, and safety

The labeled indication of the therapy was a statistically significant driver of utility for two of the four product profiles.

- Product One, indicated for B-cell precursor ALL, Large B-cell Lymphoma (LBCL), and Follicular Lymphoma (FL), achieved the highest utility among indications (U = 24.12; 95% CI: 8.99 to 39.24; p = 0.0027).
- Product Four, indicated for Relapsed/Refractory Multiple Myeloma (RRMM), also yielded a significant positive utility (U = 11.03; 95% CI: 2.78 to 19.28; p = 0.0104).

Regarding clinical efficacy, Product Two received the highest mean preference rating (7.11 ± 1.84) based on its profile of 58% Complete Response (CR) and 83% Overall Response Rate (ORR); however, the derived utility for this attribute was not statistically significant (U = −1.59; p = 0.7563).

Safety profiles did not reach statistical significance as independent drivers of utility in this model. Product One, characterized by the highest incidence of severe adverse events (32% Grade 3 CRS; 41.5% ICANS), received the lowest safety rating (3.78 ± 2.34) and a notable negative utility value (U = −9.15; 95% CI: 25.89 to 7.59; p = 0.2737), though this finding was not statistically significant.

### Economic attributes: agreements and pricing

The base price per treatment, ranging from $255,000 (Product Four) to $401,000 (Product Three), did not yield statistically significant utility values for any product profile (p > 0.05 for all pricing attributes). However, specific Managed Entry Agreements (MEAs) were significant determinants of value.

### Outcome-based agreements (OBA)

Product Three featured a performance-linked coverage model where payment is split 50/50, with the second tranche conditional on 80% of the treated cohort sustaining a response at 18 months. This attribute generated the highest utility value observed in the study (U = 24.07; 95% CI: 15.01 to 33.13; p < 0.0001). Other OBA models (individual level performance-linked) assessed for Products One, Two, and Four did not reach statistical significance (p > 0.05).

### Financial-based agreements (FBA)

- Product One, offering free treatment for one patient for every 10 patients treated, yielded a significant positive utility (U = 16.53; 95% CI: 5.87 to 27.20; p = 0.0035).
- Product Four, offering a flat 15% discount regardless of volume, also achieved statistical significance (U = 14.37; 95% CI: 6.19 to 22.56; p = 0.0011).
- Product Two, which committed to coverage by the pharmaceutical company after an annual threshold of 25 patients, showed a significant utility (U = 10.11; 95% CI: 2.17 to 18.04; p = 0.0142).

### Structural attributes: patient support and technology transfer

Non-clinical support mechanisms significantly influenced expert preferences, particularly for Product One.

- Patient Support Programs (PSP): The comprehensive PSP offered by Product One (including transportation, accommodation, and financial aid) was a significant positive driver (U = 17.11; 95% CI: 7.69 to 26.54; p = 0.0008). In contrast, PSPs focused solely on educational materials (Product Two) or psychological support (Product Three) did not yield significant utility.
- Technology Transfer: Commitments to establish local manufacturing sites were valued significantly by the panel.
o Product One (site establishment contingent on 60+ patients/year) yielded a utility of 11.60 (95% CI: 4.49 to 18.71; p = 0.0022).
o Product Three (site establishment contingent on 230+ patients/year) yielded a utility of 7.79 (95% CI: 0.355 to 15.23; p = 0.0406).
o Product Two (site establishment contingent on 280+ patients/year) yielded a utility of 8.23 (95% CI: 0.34 to 16.11; p = 0.0414).
o Product Four, which offered “Scientific Guidance Only” without a manufacturing commitment, did not achieve a significant utility score (U = −1.24; 95% CI: 9.82 to 7.33; p = 0.7698).
- Institutional Support: Attributes related to staff training and adverse event management support (Institutional Support) did not reach statistical significance for any of the four products (p > 0.05).

### Relative importance of attributes

**FIGURE 1:**
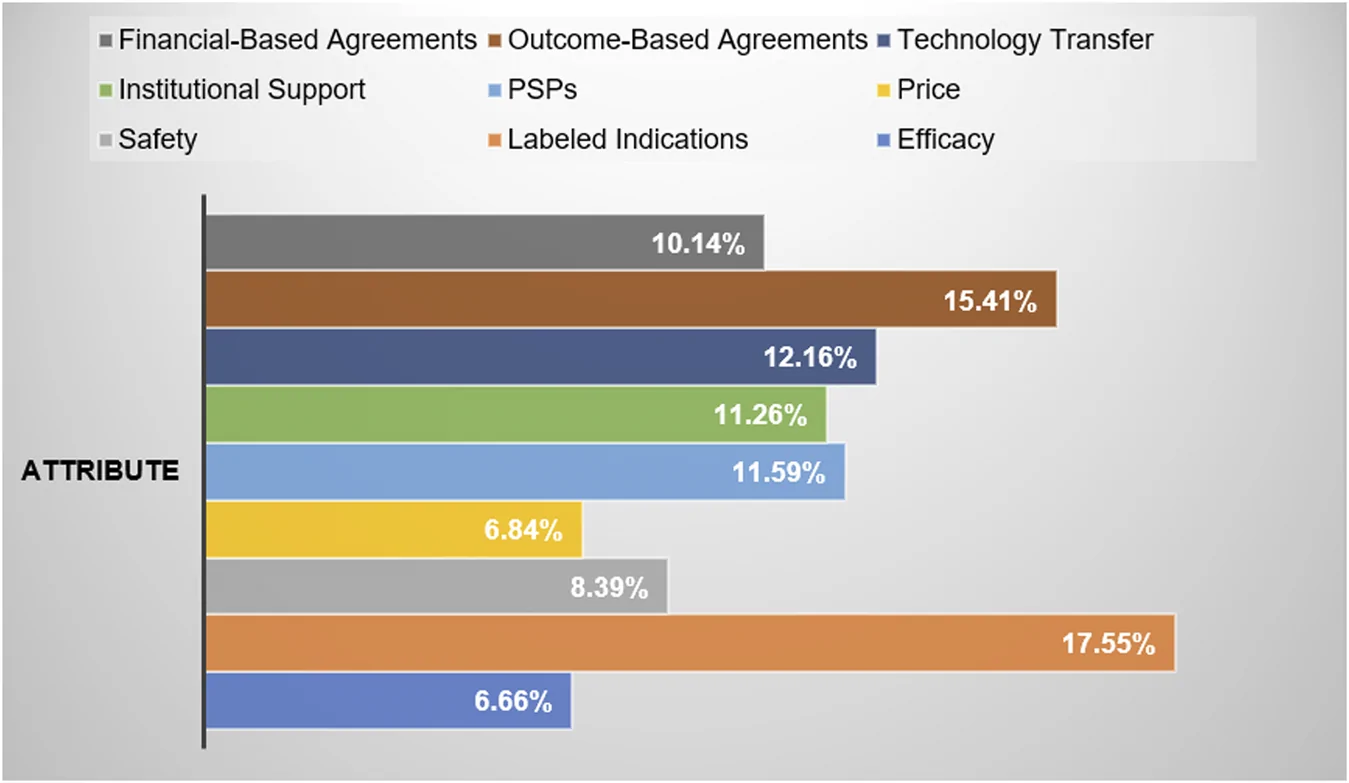
Model-derived relative attribute importance, calculated from the range of part-worth utilities and normalized to 100%. *Please note that these aggregate values are conditional on the tested levels and may obscure individual variation among the 18 participating KOLs.

Figure 1 illustrates the relative importance of various attributes in experts’ decision-making processes, informed by a range of utility estimates. The results indicate that Labeled Indications and OBAs are the primary drivers of value, contributing significantly to the overall utility weight. They are closely followed by PSPs and FBAs, which, together, outweigh other economic and structural considerations. Technology Transfer capabilities have some significance within the decision hierarchy. In contrast, attributes such as Efficacy, Safety, Price, and Institutional Support, although clinically relevant, appear less significant in this particular context, as their individual utility values did not achieve statistical significance.

## Discussion

This study quantitatively assessed the utility and relative importance of key decision-making attributes for CAR T-cell therapies, utilizing CAR T-cell therapy as a case study within the GCC region. The findings indicate that KOLs prioritize labeled indications and OBAs over upfront price and simple safety metrics. Rather than serving as a definitive roadmap, these results provide foundational empirical insights that can inform the development of a unified, value-driven framework for adopting CAR T-cell therapies in the Gulf.

### Drivers of value: indications and efficacy

The study identified labeled indication as a significant driver of utility, with Product One achieving the highest utility. The inclusion of labeled indications as a distinct attribute was driven by the rapid, real-world label expansion of CAR T-cell therapies. Health systems find additional value and operational advantages from products that can treat multiple patient populations under a single procurement framework (Yi et al., 2025). Notably, Product Four, indicated for Relapsed/Refractory Multiple Myeloma (RRMM), also yielded significant utility despite the study’s primary focus on DLBCL profiles. A possible interpretation of this finding is that it reflects the perceived high unmet need in Multiple Myeloma and the clinical benefits of BCMA-targeted therapies (Yi et al., 2025), though our model does not explicitly confirm this specific rationale.

Interestingly, clinical efficacy (response rates) and safety (CRS/ICANS rates) did not reach statistical significance as independent utility drivers in our regression model. This contrasts with traditional Choice-Based DCE frameworks where clinical benefit is often the most important criterion (Gonçalves, 2025). We hypothesize that in the specific context of CAR T-cell therapy, where the baseline efficacy is already perceived as curative or vastly superior to the standard of care, decision-makers in the GCC prioritize the mechanisms of access and affordability over incremental clinical differences between theoretical products. When baseline efficacy is perceived to be high relative to existing treatment options, the uncertainty regarding long-term budget impact shifts the decision-making focus toward coverage mechanisms, such as OBAs, rather than static clinical data (Cao et al., 2025).

### Economic attributes: value-based agreements and affordability

A pivotal finding is the high utility assigned to OBAs, particularly the cohort-level model (Product Three), which generated the highest utility value (U = 24.07). This underscores a strong preference for risk-sharing models that link reimbursement to real-world therapeutic performance (Hamad et al., 2025; Al-Omar et al., 2025). Global literature increasingly advocates for such “performance-linked managed entry agreements” to address the affordability and uncertainty of high-cost advanced therapies (Gonçalves, 2025; Tripathi et al., 2025). In the GCC, where healthcare systems are transitioning from volume-based to value-based procurement (Hamad et al., 2025; AlRuthia et al., 2025). OBAs offer a mechanism to facilitate market access without unsustainable upfront budget impacts. The preference for cohort-level over individual-level agreements likely reflects a desire for administrative simplicity, addressing the “structural barriers” and lack of integrated tracking systems often cited as hurdles in the region (Abu-Shraie et al., 2023).

To provide broader context on these financing mechanisms, recent literature highlights the potential role of multi-stakeholder collaboration, robust data infrastructure for outcomes measurement, and innovative contracting as tools that may help address clinical uncertainty and short-term budget impact when appropriate (Phares et al., 2024; Wagner et al., 2024; Wagner et al., 2026). Although these studies focus primarily on gene therapies rather than CAR T-cell therapies, several underlying considerations-including managed entry agreements, budget-impact mitigation, outcomes measurement, and value assessment-may be relevant to the GCC context. Gene therapies may use either *in vivo* or *ex vivo* approaches, whereas currently available CAR T-cell therapies are *ex vivo*, gene-modified cellular therapies. Accordingly, differences in target diseases, patient populations, manufacturing requirements, administration pathways, institutional readiness, monitoring, and toxicity management should be considered when adapting these frameworks. Gene therapy contracting models may therefore provide useful precedent, but CAR T-cell agreements should be tailored to the specific clinical, logistical, and infrastructure requirements of these therapies.

### Accessibility and structural challenges in the GCC

The study highlights the critical importance of structural attributes, specifically PSPs and Technology Transfer. A comprehensive PSP (Product One) was a significant positive driver, indicating that decision-makers value holistic support that reduces out-of-pocket burdens and logistical barriers. Moreover, the commitment to establish local manufacturing sites was significantly valued over “Scientific Guidance Only.” This reflects a strategic desire for regional health security and operational resilience, aligning with recent literature emphasizing that decentralized and hospital-based manufacturing is key to mitigating bottlenecks and improving global access to CAR T-cell therapies.

To translate these expert preferences into actionable policy, GCC healthcare systems may need to address several foundational gaps. First, the region requires robust legal frameworks enabling OBAs, which are currently fragmented. Second, the preferred cohort-level agreements necessitate the urgent establishment of a unified registry infrastructure for outcome tracking. Third, decision-makers must implement explicit technology transfer incentives that de-risk the investment required for local manufacturing.

### Strengths and limitations

A notable strength of this study lies in its use of a Rating-Based Conjoint Analysis (RBCA). While distinct from traditional Discrete Choice Experiments (DCEs) that rely on choice probabilities, this metric compositional approach allowed respondents to systematically evaluate highly complex, multifaceted attributes without succumbing to cognitive overload (Arroyo et al., 2017; Asioli et al., 2016). This enabled the identification of specific value drivers, such as the distinct preference for cohort-based OBAs. However, the study has limitations. The sample size was relatively small (n = 18), which, while typical for expert panels, restricts generalizability. Additionally, while Saudi Arabia is an acknowledged regional leader in successfully executing OBAs, Saudi experts were underrepresented in our panel (n = 1) (AlRuthia et al., 2025; Abu-Shraie et al., 2023). This underrepresentation is a limitation, as the aggregate preferences may lean heavily toward the perspectives of GCC nations that are still in the nascent stages of adopting risk-sharing frameworks, rather than reflecting the region’s most mature OBA markets. Moreover, although standard OLS regression effectively identified directional value drivers, it did not incorporate clustered standard errors or mixed-effects modeling to address within-respondent correlation across multiple profiles evaluated by each expert. Future research should build on these findings through larger-scale choice-based discrete choice experiments in larger cohorts, using appropriate repeated-measures modeling to evaluate the robustness and feasibility of the identified preference patterns.

## Conclusion

Decision-makers in the GCC prioritize labeled indications and risk-sharing Outcome-Based Agreements when evaluating high-cost CAR T-cell therapies. To support equitable and sustainable access, GCC healthcare systems may consider advancing VBHC frameworks that enable appropriate innovative contracting and investment in local infrastructure.

## Data Availability

The original contributions presented in the study are included in the article/supplementary material, further inquiries can be directed to the corresponding author.
